# High Intra- and Inter-Tumoral Heterogeneity of *RAS* Mutations in Colorectal Cancer

**DOI:** 10.3390/ijms17122015

**Published:** 2016-12-01

**Authors:** Marion Jeantet, David Tougeron, Gaelle Tachon, Ulrich Cortes, Céline Archambaut, Gaelle Fromont, Lucie Karayan-Tapon

**Affiliations:** 1 Faculté de Médecine Pharmacie, Université de Poitiers, 86021 Poitiers, France; marion_doc@hotmail.fr (M.J.); david.tougeron@chu-poitiers.fr (D.T.); gaelle.tachon@chu-poitiers.fr (G.T.); ulrich.cortes@chu-poitiers.fr (U.C.); labonco@gmail.com (C.A.); 2 Département de Cancérologie Biologique, Centre Hospitalo-Universitaire de Poitiers, 86021 Poitiers, France; 3 Département d’anatomopathologie, Centre Hospitalo-Universitaire de Poitiers, 86021 Poitiers, France; Gaelle.fromont-hancard@univ-tours.fr; 4 Département de Gastroentérologie, Centre Hospitalo-Universitaire de Poitiers, 86021 Poitiers, France; 5 Laboratoire Inflammation, Tissus Epithéliaux et Cytokines, EA 4331, Université de Poitiers, 86021 Poitiers, France; 6 INSERM1084, Laboratoire de Neurosciences Expérimentales et Cliniques, Université de Poitiers, 86021 Poitiers, France

**Keywords:** colorectal cancer, *RAS* mutation, intra-tumoral heterogeneity, inter-tumoral heterogeneity

## Abstract

Approximately 30% of patients with wild type *RAS* metastatic colorectal cancer are non-responders to anti-epidermal growth factor receptor monoclonal antibodies (anti-EGFR mAbs), possibly due to undetected tumoral subclones harboring *RAS* mutations. The aim of this study was to analyze the distribution of *RAS* mutations in different areas of the primary tumor, metastatic lymph nodes and distant metastasis. A retrospective cohort of 18 patients with a colorectal cancer (CRC) was included in the study. Multiregion analysis was performed in 60 spatially separated tumor areas according to the pathological tumor node metastasis (pTNM) staging and *KRAS*, *NRAS* and *BRAF* mutations were tested using pyrosequencing. In primary tumors, intra-tumoral heterogeneity for *RAS* mutation was found in 33% of cases. Inter-tumoral heterogeneity for *RAS* mutation between primary tumors and metastatic lymph nodes or distant metastasis was found in 36% of cases. Moreover, 28% of tumors had multiple *RAS* mutated subclones in the same tumor. A high proportion of CRCs presented intra- and/or inter-tumoral heterogeneity, which has relevant clinical implications for anti-EGFR mAbs prescription. These results suggest the need for multiple *RAS* testing in different parts of the same tumor and/or more sensitive techniques.

## 1. Introduction

Colorectal cancer (CRC) is the third deadliest of all cancers [1]. Nearly one-third of the patients will eventually die of the disease. Targeting the epidermal growth factor receptor (EGFR), an important component in CRC carcinogenesis, is one of the major therapeutic options in metastatic CRC (mCRC). Two anti-EGFR monoclonal antibodies (mAbs), cetuximab and panitumumab, are commonly used in mCRC. Clinical trials have shown the benefit of anti-EGFR mAbs alone or in combination with chemotherapy in mCRC [2,3,4].

Several studies have demonstrated that *KRAS* mutation in exon 2 is a predictive marker of resistance to anti-EGFR mAbs [5]. More recently, other activating *RAS* mutations (*KRAS* exons 3 and 4 and *NRAS* exons 2, 3 and 4) were also shown to confer resistance to anti-EGFR mAbs [3,4]. Approximately 50% of mCRC harbor mutations in exons 2, 3 or 4 of either *KRAS* or *NRAS* genes [6]. The most frequent mutations are detected in exon 2 (codons 12 and 13) of *KRAS* (40%), and, to a lesser extent, in exon 3 (codons 59 and 61) and exon 4 (codons 117 and 146) of *KRAS* (≈7% of cases). Activating mutations of *NRAS* occur only in a subset of mCRC (≈5% of cases), mostly at codons 12, 13 and 61 [6]. The *BRAF^V600E^* mutation occurs in 10%–15% of mCRC [7,8]. *BRAF^V600E^* mutant mCRC is associated with poorer outcomes. However, whether this mutation is predictive of resistance to anti-EGFR mAbs is uncertain [7].

Only wild-type (WT) *RAS* mCRCs benefit from treatment with anti-EGFR mAbs. Nevertheless, nearly 35% of patients with WT *RAS* tumors do not respond to anti-EGFR treatment [3,4,6]. Several molecular mechanisms underlying the development of treatment resistance have been reported in the literature [9]. One possible explanation lies in tumor heterogeneity with regard to *RAS* mutations [8,10]. There is a general consensus that progression of cancer develops from a single mutated cell, followed by clonal expansion associated with genetic alterations. The acquisition of these alterations can result in the emergence of new tumor subclones with different genotypes [11]. Intra-tumoral heterogeneity is defined by the presence of at least two different tumoral subclones within the same tumor mass. Inter-tumoral heterogeneity consists in the presence of at least two different tumor subclones at different tumor sites in a single patient (i.e., primary tumor, metastatic lymph nodes or metastases) [12]. Both intra- and inter-tumoral heterogeneity are important to identify since they could affect response to targeted therapies.

Different levels of tumoral heterogeneity have already been observed in several tumor types [13,14,15]. Nevertheless, there are few data concerning intra- and inter-tumoral heterogeneity in CRC. *KRAS*, *NRAS* and *BRAF* mutations are considered to be mutually exclusive in CRC [16]. Inter-tumoral heterogeneity seems to be relatively low between primary and metastatic lesions in mCRC since concordance of *KRAS* and *BRAF* status is over 95% [17,18,19]. Nevertheless, these previous works used sequencing methods with low sensitivity and did not study complete *RAS* status. In addition, few data have been available concerning inter-tumoral heterogeneity of *RAS* and *BRAF* mutations between primary tumors and lymph node metastasis. Data concerning intra-tumoral heterogeneity of *RAS* and *BRAF* mutations between different areas of primary tumor data are lacking. In the present study, we investigated intra- and inter-tumoral heterogeneity of *RAS* and *BRAF* mutations in 60 tumor areas from 18 CRCs.

## 2. Results

### 2.1. Population

We retrospectively analyzed tumors from 18 patients with CRC (twelve colons and six rectums). Mean age at diagnosis was 66.5 ± 9.0 years (Table 1). Tumor stages were stage I (*n* = 1, 5%), stage II (*n* = 3, 17%), stage III (*n* = 5, 28%) and stage IV (*n* = 9, 50%). According to the pathological tumor node metastasis (pTNM) staging, tumors were pT2 (*n* = 1, 5%), pT3 (*n* = 14, 78%) and pT4a (*n* = 3, 17%).

Tumors were assessed for *KRAS* mutations in codons 12 and 13 (*KRAS*^12−13^) on the initial transparietal section (ITS). Five tumors were *KRAS*^12−13^ mutant (MUT), seven tumors were *KRAS*^12−13^ WT and six tumors had potential low level (PLLM) *KRAS*^12−13^ mutations (Table 2). Tumors with PLLM are defined as tumors with mutant allele frequency ≥limit of detection (LOD) and ≤LOD + 3% as described in the materials and methods section [8]. One WT *KRAS* tumor had a *BRAF^V600E^* mutation.

### 2.2. Intra-Tumoral and Inter-Tumoral Heterogeneity of KRAS and NRAS Mutations

Thirty-nine percent of mCRC studied harbored intra- and/or inter-tumoral heterogeneity (Table 2).

Among the six tumors with intra-tumoral heterogeneity, only two were *KRAS*^12−13^ WT in ITS (cases 2 and 4). Three tumors (cases 2, 7, 12) presented *KRAS*^12−13^ PLLM or MUT in at least one of the tumor areas selected, but they also presented other *KRAS* or *NRAS* MUT or PLLM in other areas of the tumor (case 2) or in the same area (case 7, 12). An additional *NRAS*^117^ PLLM in the submucosa (pT1) was observed for one tumor (case 18) (Table 2 and Figure S1). All in all, 33% of tumors (6/18) showed intra-tumoral heterogeneity for *RAS* mutation. The others presented either a WT pattern (cases 1, 3, 5, 6 and 10) or the same mutation with variation in allele frequency (cases 8, 11, 13–17) for all intra-tumoral areas (Table 2 and Table S1).

To evaluate the inter-tumoral heterogeneity, we tested the metastatic lymph nodes and distant metastases for *KRAS*, *NRAS* and *BRAF* mutations when available. Among 11 cases analyzed, four presented inter-tumoral heterogeneity (36%). Three mutated primary tumors were WT in the metastatic lymph nodes (N+) or in the visceral metastases (M+) (cases 2, 12, 14) (Table 2).

It is worth noting that all patients with mCRC with *RAS* PLLM and/or *RAS* mutation in a limited area of the tumor (i.e., intra- or inter-tumoral heterogeneity) treated with anti-EGFR mAbs had disease progression at 2–3 months (*n* = 5, cases 2, 7, 9, 11 and 12).

### 2.3. Mutational Intra-Tumoral Heterogeneity

**Figure 1:**
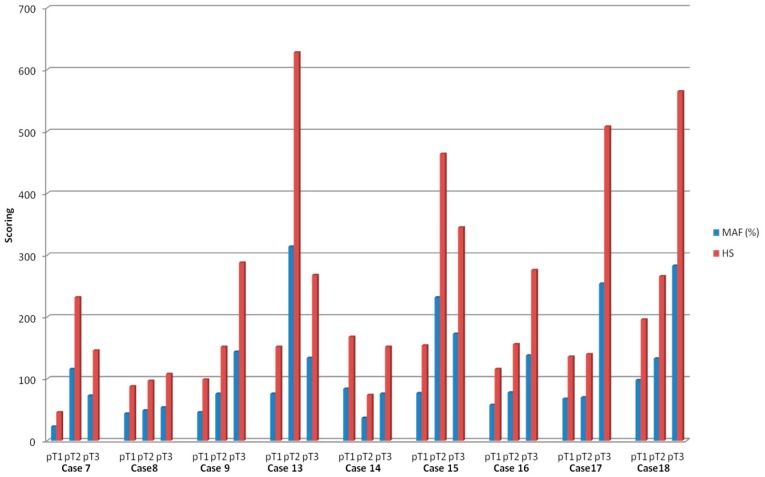
Mutation allele frequency and heterogeneity score. Variability of mutation allele frequency and heterogeneity score between tumoral zone selections in cases which harbored the same mutation in pT1 to pT3. MAF: mutation allele frequency; HS: heterogeneity score.

We focus on mutational intra-tumoral heterogeneity, defined as subclones with different mutant allele frequencies. Therefore, we calculated mutant allele frequency in neoplastic cells (MAFnc) and heterogeneity score (HS) for cases 7–9 and 13–18, which harbored the same mutation from the submucosa (pT1), the muscular layer (pT2) to the subserosa (pT3) (Figure 1). MAFnc is the mutant allele frequency normalized to 100% tumoral cells and HS corresponds to the fraction of neoplastic cells carrying a specific mutation and was calculated assuming that somatic mutations are usually heterozygous events [20]. Mean HS increased with T stage, 128.4 ± 46.1 standard deviation (SD) in pT1, 234.1 ± 186.8 SD in pT2 and 296.2 ± 156.9 SD in pT3 (*p* = 0.03) (Figure S2). We observed that HS was higher in pT3 areas in 55% of cases (*n* = 5/9) regardless of the mutation. Mean total HS (sum of HS for each mutation in one tumor) was 692.6 ± 262.6 SD. Total HS score was associated neither with tumor stage (*p* = 0.78), nor with tumor location (*p* = 0.90), nor with tumor recurrence (*p* = 0.56).

### 2.4. RAS Mutations Are Not Exclusive

*RAS* mutations are considered to be mutually exclusive. Only a few articles have reported cases of coexisting *KRAS* and *NRAS* mutations [21]. However, in our study, we observed that different *KRAS* mutations as well as *KRAS* and *NRAS* mutations may coexist. Five tumors had multiple *RAS* mutated subclones in the same tumor (28%). Moreover, four presented, in one of their tumor selections, at least two *RAS* mutated clones (22%) (cases 7, 9, 12, and 18) (Table 2). Indeed, we observed the coexistence of different *KRAS* mutations in three tumors (17%) (cases 7, 9, 12) as well as *KRAS* and *NRAS* mutations in three tumors (17%) (cases 7, 9 and 18). 

Tumors with two or more *RAS* mutations (cases 2, 7, 9, 12, and 18) as compared with tumors showing one *RAS* mutation (cases 8, 11, 13 to 17) were associated neither with tumor stage (*p* = 0.29) nor with tumor recurrence (*p* = 0.07).

## 3. Discussion

This study revealed a high proportion of intra- and inter-tumoral heterogeneity for *RAS* mutations in metastatic colorectal cancer. Nearly 40% of the mCRC studied harbored intra- and/or inter-tumoral heterogeneity. We also demonstrated the coexistence of different *RAS* mutations within the same tumor. These results have relevant clinical implications for anti-EGFR monoclonal antibodies prescription in mCRC since *RAS* mutations confer resistance to this treatment. Hence, testing of *KRAS* and *NRAS* mutations (codons 12, 13, 59, 61, 117 and 146) is a prerequisite for anti-EGFR mAbs used in mCRC. In daily practice, this testing usually relies on a single tumor sample with high tumor cell content. However, some patients with *RAS* WT tumor have primary resistance to anti-EGFR mAbs. One explanation is the limited sensitivity of testing methods, leading to false negative results [6,8,10]. Nevertheless, intra- and/or inter-tumoral heterogeneity of *RAS* mutations could be the most important cause of therapeutic failure.

Indeed, recently, the “Big Bang” model emphasized that clonal alterations and subclonal events are an early event in CRC carcinogenesis with subclone mixing [22]. Several neoplastic subclones with co-existing mutations in different genes (as well as different molecular alterations) could be present in a single primary tumor with different mutant allele frequencies [19,20,23,24,25]. In our study, by testing *KRAS* and *NRAS* mutations in histologically relevant macrodissected zones according to pTNM, we observed the presence of (i) mutational intra-tumor heterogeneity with at least two co-existing *KRAS* and/or *NRAS* mutations within the same tumor areas and (ii) spatial intra-tumoral heterogeneity with coexistence within the same tumor of *KRAS* and/or *NRAS* mutated zones and WT zones. This intra-tumoral heterogeneity was found in 33% of cases in our study. Kosmidou et al. reported similar results (44%) of intra-tumoral heterogeneity for *KRAS* mutations when they compared tumor center and tumor periphery [23]. Recently, Kim et al. observed a substantial level of intra-tumoral heterogeneity (46% to 80%) on multiregion biopsies from five mCRCs [26]. Up until now, our study is one of the largest study concerning intra-tumoral heterogeneity and the only one concerning an extended *RAS* status.

*KRAS*, *NRAS* and *BRAF* mutations are considered to be mutually exclusive in CRC [16]. Nevertheless, in our study, we observed the coexistence of two different *RAS* mutations in 28% of cases. We identified the coexistence of different *KRAS* mutations as well as *KRAS* and *NRAS* mutations. Coexistence of different *KRAS*^12−13^ mutations has been reported by others on small series [24,25,26]. To our knowledge, the present study is one of the first reports concerning the coexistence of mutations in codons 12–13, 61 and 146 of *KRAS* (17% of cases). In addition, we demonstrated the coexistence of mutations in *KRAS* and *NRAS*, which has been reported only once in Vagaja, N.’s article [21].

**Figure 2:**
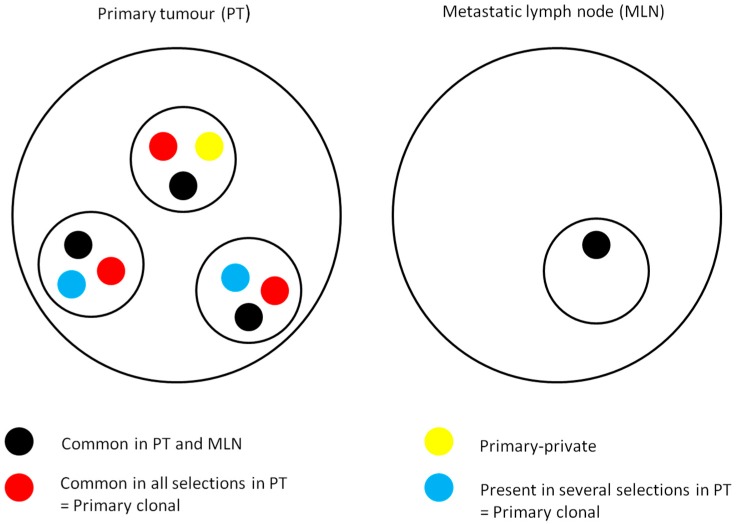
Subclone distribution in primary tumor and metastatic lymph node. In some cases, *KRAS* or *NRAS* mutations were universal/common as they were present in all macrodissected regions in primary tumors (PTs) and in metastatic lymph nodes (MLNs). In other cases, we observed multiple subclones with mutations present only in several regions but not all, or present in all regions in PT but not in MLN, and they could be classified as primary-clonal. In other cases, mutation was present only in one area of the tumor and could be classified as primary-private.

Concordance of *KRAS* and *BRAF* status between primary and metastatic lesions in mCRC has been considered to be over 95% [17,18,19]. To our knowledge, few studies have evaluated inter-tumoral heterogeneity of *RAS* mutations between primary tumors and lymph nodes or distant metastatic lesions. We observed 36% of inter-tumoral heterogeneity, with *RAS* mutated primary tumors being WT in lymph nodes and/or distant metastatic lesions. In addition, metastatic lymph nodes may have different *RAS* mutations as compared to primary tumors. Concerning metastatic lymph nodes, only limited data concerning *KRAS* mutations are available. In one study, heterogeneity in *KRAS* mutations between primary tumors and lymph node metastases was found in approximately 30% of cases [27]. To summarize, in some tumors, *KRAS* or *NRAS* mutations were “universal” as they were present in all macrodissected areas in primary lesions and in metastases. In other cases, we observed multiple subclones with mutations present only in one area or present in all areas in primary lesions but not in metastases, and they could be classified as “primary-private” and as “primary-clonal”, respectively, as described by others [26] (Figure 2).

In daily practice, *RAS* mutation testing is usually carried out on a single transparietal section of a given location with a high fraction of neoplastic cells. However, our results have shown that not all *RAS* mutations present in the tumor can be detected by the current sampling method. One alternative suggestion might be the preparation of a DNA mix obtained after macrodissection of multiple histologically relevant areas, as previously proposed [28]. Moreover, for patients with multiple tumor biopsies and/or surgeries, it is important to perform *RAS* analyses on each sample. In addition, all *KRAS* and *NRAS* mutations must be sought simultaneously in first-line testing as they are not mutually exclusive.

All of these results concerning intra- and inter-tumoral heterogeneity are dependent on the sensitivity of the technique used. The more sensitive the sequencing technique, the higher the probability of detecting minority subclones when assessing intra- and inter-tumoral heterogeneity [8,10,29]. However, the clinical relevance of these minority subclones called “potentially low-level mutants” is unknown (i.e., impact on anti-EGFR mAbs resistance). In a previous work, we demonstrated that low-frequency *KRAS* mutations (2.3% to 10%) are associated with resistance to anti-EGFR mAbs [8]. More recently, Laurent-Puig et al., using highly sensitive picodroplet digital PCR (detection of one mutant *KRAS* allele in 200,000 WT *KRAS* alleles), suggested that patients with mCRC showing *KRAS*-mutated subclones lower or equal to 1% were benefiting from anti-EGFR therapies, while others were not (i.e., >1%) [10]. Due to the limited number of patients treated with anti-EGFR mAbs in our series, we cannot reliably assess the impact of intra- and inter-tumoral heterogeneity on anti-EGFR mAbs efficacy. Nevertheless, all patients with a mCRC harboring *RAS* PLLM and/or *RAS* mutations in a limited part of the tumor (i.e., intra- or inter-tumoral heterogeneity), and treated with anti-EGFR mAbs showed disease progression (*n* = 5). To conclude, *RAS* mutated subclones (>1%) partially explain primary anti-EGFR mAbs resistance. In contrast, secondary resistances to anti-EGFR mAbs are partially due to *RAS* and *EGFR* mutation [30,31,32].

One limitation of our study is the limited number of patients included, even though 78 samples from 18 tumors were analyzed. Moreover, in some cases, PPLM was found only in ITS but not in selected macrodissected areas in primary tumors. Interestingly, these results involved high tumoral cellularity cases, thereby confirming that the mutation is present but remains undetectable in the selected macrodissected areas. The major strengths of this study are: (1) analysis of extended *RAS* status; (2) multiple testing in primary tumors, metastatic lymph nodes and metastasis; and (3) use of a highly sensitive method.

With regard to tumor infiltration, the mutations were more frequently found in subserosa selections (pT3) and heterogeneity score increased with wall invasion; thus, pT3 selection seemed to be the best sampling zone for molecular analysis. In addition, we observed that HS was very high in several areas with a low fraction of neoplastic cells, thereby suggesting possible bias due to low tumoral cellularity or genomic amplification of the mutant allele or loss of the wild-type allele as well as mutation in both alleles [33].

## 4. Materials and Methods

### 4.1. Tumor Samples

**Figure 3:**
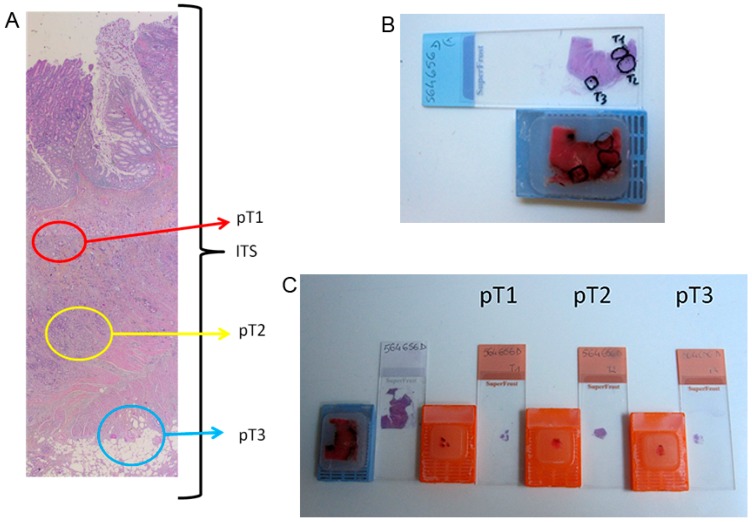
Tumoral selection and macrodissection. (**A**) Tumoral selections on the HES slide: tumoral selections on the HES slide were performed according to pathological tumor node metastasis staging (pTNM) 2009 in submucosa (pT1), in the muscular layer (pT2) for pT2 tumors, selection in submucosa, in the muscular layer and in subserosa (pT3) for pT3 and pT4 tumors; (**B**) tumoral selections on the HES slide and paraffin block: tumoral zones selected on the HES slide were transposed on the corresponding paraffin block; and (**C**) paraffin blocks and HES slides of macrodissected tumoral selections: the corresponding area of each tumoral selection was macrodissected and included in a new paraffin block. An additional HES staining was performed to ensure correspondence with the originally selected area. ITS: initial transparietal section; HES: haematoxylin-erythrosin-saffron.

Tumoral zone selection was performed under HES (Haematoxylin-Erythrosin-Saffron) staining by two experienced pathologists (Marion Jeantet and Gaelle Fromont) according to the 2009 pTNM classification [34]. For each tumor, selections involved the submucosa (pT1), the muscular layer (pT2), the subserosa (pT3), the metastatic lymph nodes (N+) and/or the visceral metastases (M+) when available. For instance, for a pT2 tumor, we performed pT1 and pT2 selections. For a pT3 or pT4 tumor, we performed pT1, pT2 and pT3 selections (Figure 3). The corresponding area of each tumoral zone selection was macrodissected and included in a new paraffin block. Another HES staining was performed to verify correspondence with the originally selected area. The percentage of tumor cells was assessed and selections with less than 5% of tumor cells were excluded.

A total of 60 tumoral macrodissections were analyzed. Among these samples, 15 were in pT1, 18 in pT2, 16 in pT3, 10 in N+ and 1 in M+. Four to six 10 µm thick sections were used for molecular analysis.

### 4.2. KRAS, NRAS and BRAF Mutation Testing

Genomic DNA from macrodissected sections was extracted with the QIAamp DNA FFPE Tissue kit (Qiagen, Hilden, Germany). The 18 initial samples and the 60 newly macrodissected samples were tested for *KRAS* and *NRAS* mutations in codons 12–13 (*KRAS*^12−13^ and *NRAS*^12−13^), codon 59 (*KRAS*^59^ and *NRAS*^59^), codon 61 (*KRAS*^61^ and *NRAS*^61^), codon 117 (*KRAS*^117^ and *NRAS*^117^) and codon 146 (*KRAS*^146^ and *NRAS*^146^). *BRAF* was tested for mutation in codon 600 (*BRAF^V600E^*). Mutations were detected by pyrosequencing using TheraScreen *KRAS* PyroKit CE-IVD kit (Qiagen) for *KRAS*^12−13^ and *KRAS*^61^ or the PyroMark PCR kit (Qiagen) with homemade primers using PyroMark Assay Design 2.0 software (Qiagen, Hilden, Germany) for other *RAS* [35] and *BRAF* mutations (Table S2). PCR was carried out using 50 ng of DNA in a total volume of 25 µL according to the Qiagen supplier’s instructions. Each series included a known-mutated and a known wild-type sample, as positive and negative controls. Pyrosequencing was performed using a Pyromark Q24 MDx according to the manufacturer’s instructions (Qiagen).

### 4.3. Determination of Mutant Allele Burden and Tumoral Heterogeneity

Mutant allele frequency was determined using PyroMark Q24 2.0.6 software (Qiagen). Limit of blank (LOB) and limit of detection (LOD) were considered during biological interpretation and all samples were analyzed with regard to the LOD, which was specific to each nucleotide position [35]. Interpretation was carried out as follows: when the mutant allele frequency was <LOD, the sample was considered as WT; when the mutant allele frequency was ≥LOD and ≤LOD + 3%, it was considered as PLLM and when the mutant allele frequency was >LOD + 3%, it was considered as MUT. All mutations (PLLM and MUT) were confirmed at least twice, in two independent experiments.

Mutant allele frequency in neoplastic cells (MAFnc) and heterogeneity score (HS) were calculated as described in Normanno et al. [20]. MAFnc is the mutant allele frequency normalized for the tumor cell content. Assuming that somatic mutations are usually heterozygous events, the HS was calculated by multiplying the frequency of mutant alleles in tumor cells by two. Therefore, HS virtually corresponds to the fraction of tumor cells carrying a specific mutation. HS > 100 indicates a copy number variation, either a gain of mutant allele or a loss of WT allele.

### 4.4. Statistical Analyses

Differences in HS according to clinicopathological parameters were analyzed using the Mann–Whitney *U* test (comparison of two groups) or Kruskal–Wallis test (comparison of more than two groups). For dichotomous variables, Fisher's exact test was used. Statistical analyses were carried out with a two-sided test with a significance value of 0.05. All analyses were performed using Statview software (Statview for Windows, SAS Institute, version 5.0, Cary, NC, USA).

## 5. Conclusions

Concordance of *KRAS* and *BRAF* status between primary and metastatic lesions in mCRC has been considered to be over 95% [17,18,19]. To our knowledge, the present study is one of the first reports concerning inter-tumoral heterogeneity of *RAS* mutations between primary tumors and lymph nodes or distant metastatic lesions. In conclusion, we have demonstrated the tumoral heterogeneity of *RAS* mutation and the co-existence of different *RAS* mutations in CRC, which may have major clinical implications. Our results question our daily practice and expose the limits of single transparietal sampling to ensure optimal molecular analysis. Macrodissection of multiple histologically relevant areas can be an interesting solution as proposed by Richman [28]. The use of new sensitive new generation sequencing methods can also be an alternative option [36]. However, the best solution may arise from peripheral blood testing since circulating tumor cells and circulating tumor DNA are the reflection of the whole tumor [37,38]. These technologies provide another avenue to detect mutations, especially in patients with lymph nodes or distant metastasis.

## Figures and Tables

**Table 1:** Patients and tumor characteristics.

Patient	Age	Sex	Tumor Site	Stage	pTNM 2009	Initial *KRAS*	Initial *BRAF*	Recurrence	OS	Status
2	77.9	F	right colon	III	pT3N1bM0	WT	WT	yes	48.68	dead
3	75.9	M	left colon	III	pT4aN2bM0	WT	WT	no	2.50	alive
4	79.2	F	right colon	II	pT3N0M0	WT	V600E	no	59.14	alive
6	55.9	M	left colon	I	pT2N0M0	WT	WT	no	62.50	alive
7	67.3	M	left colon	III	pT3N1bM0	G12S	WT	yes	69.21	dead
8	72.9	F	left colon	IV	pT3N1bM1	G12V	WT	-	26.35	dead
9	58.5	M	left colon	III	pT4aN0M0	G12S	WT	yes	82.86	dead
12	77.8	F	left colon	IV	pT4aN0M1	G12D	WT	-	22.60	dead
14	56.4	F	right colon	IV	pT3N1aM1	G12V	WT	-	48.39	dead
15	77.7	M	left colon	IV	pT3N2bM1	G12D	WT	-	14.41	dead
16	74.7	F	right colon	II	pT3N0M0	G12V	WT	no	37.70	alive
13	66.0	M	left colon	II	pT3N0M0	G12V	WT	-	49.00	alive
1	61.9	M	rectum	IV	pT3N2bM1	WT	WT	-	22.5	dead
5	58.9	M	rectum	IV	pT3N0M1	WT	WT	-	52.34	dead
10	57.1	F	rectum	IV	pT3N2bM1	WT	WT	-	44.80	dead
11	65.2	F	rectum	IV	pT3N2bM1	G12D	WT	-	17.96	dead
17	54.9	F	rectum	III	pT3N2aM0	G12V	WT	yes	41.22	alive
18	58.6	M	rectum	IV	pT3N0M1	G12R	WT	-	43.82	dead

M: male; F: female; OS: overall survival; WT: wild-type; pTNM: pathological tumor node metastasis.

**Table 2:** *RAS* and *BRAF* mutations in the initial transparietal sections (*n* = 13) and spatially separated tumor areas (*n* = 43) in 13 colorectal cancers.

Heterogeneity	Patients	pTNM	%TC	*KRAS*	Other Genes
Intra-tumoral heterogeneity	4	ITS	70	WT	*BRAF*:V600E (PLLM)
		pT1	30	WT	WT
		pT2	90	WT	*BRAF*:V600E (MUT)
	9	ITS	70	G12S (PLLM)/Q61R (PLLM)	*NRAS*:Q61R (MUT)
		pT1	95	WT	*NRAS*:Q61R (MUT)
		pT2	70	WT	*NRAS*:Q61R (MUT)
		pT3	25	WT	*NRAS*:Q61R (MUT)
	18	ITS	80	G12R (MUT)	WT
		pT1	30	G12R (MUT)	*NRAS* : K117N (PLLM)
		pT2	35	G12R (MUT)	WT
		pT3	20	G12R (MUT)	WT
Intra-tumoral and Inter-tumoral heterogeneity	2	ITS	80	WT	*NRAS*: A59T (PLLM)
		pT2	15	WT	WT
		pT3	20	G13D (MUT)	WT
		N	5	WT	WT
	7	ITS	60	G12S (PLLM)	FA (*NRAS*61 et *KRAS*146)
		pT1	70	G12D (MUT)/Q61L (PLLM)	*NRAS*:Q61K (MUT)
		pT2	25	G12S (MUT)	*NRAS*:Q61K (MUT)
		pT3	40	Q61L (PLLM)	*NRAS*:Q61K (MUT)
		N	25	WT	*NRAS*:Q61K (MUT)
	12	ITS	40	G12D (PLLM)/A146T (MUT)	WT
		pT2	15	A146T (MUT)	WT
		pT3	20	Q61H(PLLM)/A146T (MUT)	FA (*BRAF*)
		M	25	WT	FA(*NRAS*59)
Inter-tumoral heterogeneity only	14	ITS	30	G12V (MUT)	WT
		pT1	5	G12V (PLLM)	WT
		pT2	40	G12V (MUT)	WT
		pT3	5	G12V (PLLM)	WT
		N	5	WT	WT
Mutation without heterogeneity	8	ITS	30	G12V (MUT)	WT
		pT1	60	G12V (MUT)	WT
		pT2	75	G12V (MUT)	WT
		pT3	60	G12V (MUT)	WT
		N	80	G12V (MUT)	WT
	11	ITS	5	G12D (PLLM)	WT
		pT2	5	G12D (MUT)	WT
		pT3	5	G12D (PLLM)	WT
		N	5	G12D (MUT)	WT
	13	ITS	70	G12V (MUT)	WT
		pT1	70	G12V (MUT)	WT
		pT2	10	G12V (MUT)	WT
		pT3	15	G12V (MUT)	WT
	15	ITS	40	G12D (MUT)	WT
		pT1	60	G12D (MUT)	WT
		pT2	15	G12D (MUT)	WT
		pT3	20	G12D (MUT)	WT
		N	40	G12D (MUT)	WT
	16	ITS	40	G12V (MUT)	WT
		pT1	80	G12V (MUT)	WT
		pT2	40	G12V (MUT)	WT
		pT3	10	G12V (MUT)	WT
	17	ITS	30	G12V (MUT)	WT
		pT1	30	G12V (MUT)	WT
		pT2	20	G12V (MUT)	WT
		pT3	10	G12V (MUT)	WT
		N	20	G12V (MUT)	WT

%TC: percentage of tumoral cells; ITS: initial transparietal section; N: metastatic lymph node; M: metastasis; WT: wild-type; PLLM: potential low level mutation; MUT: mutant; FA: failed analysis.

## Supplementary Materials

Supplementary materials can be found at www.mdpi.com/1422-0067/17/12/2015/s1.
