# Supplementary material for: High Intra- and Inter-Tumoral Heterogeneity of RAS Mutations in Colorectal Cancer
Source: Int J Mol Sci. 2016 Dec 1;17(12):2015. doi: 10.3390/ijms17122015 (PMC5187815; doi:10.3390/ijms17122015)
Supplement: Supplementary file 1 [file ijms-17-02015-s001.pdf]

# Supplementary Materials: High Intra- and Inter-Tumoral Heterogeneity of *RAS* Mutations in Colorectal Cancer

**Marion Jeantet, David Tougeron, Gaelle Tachon, Ulrich Cortes, Céline Archambaut, Gaelle Fromont and Lucie Karayan-Tapon**

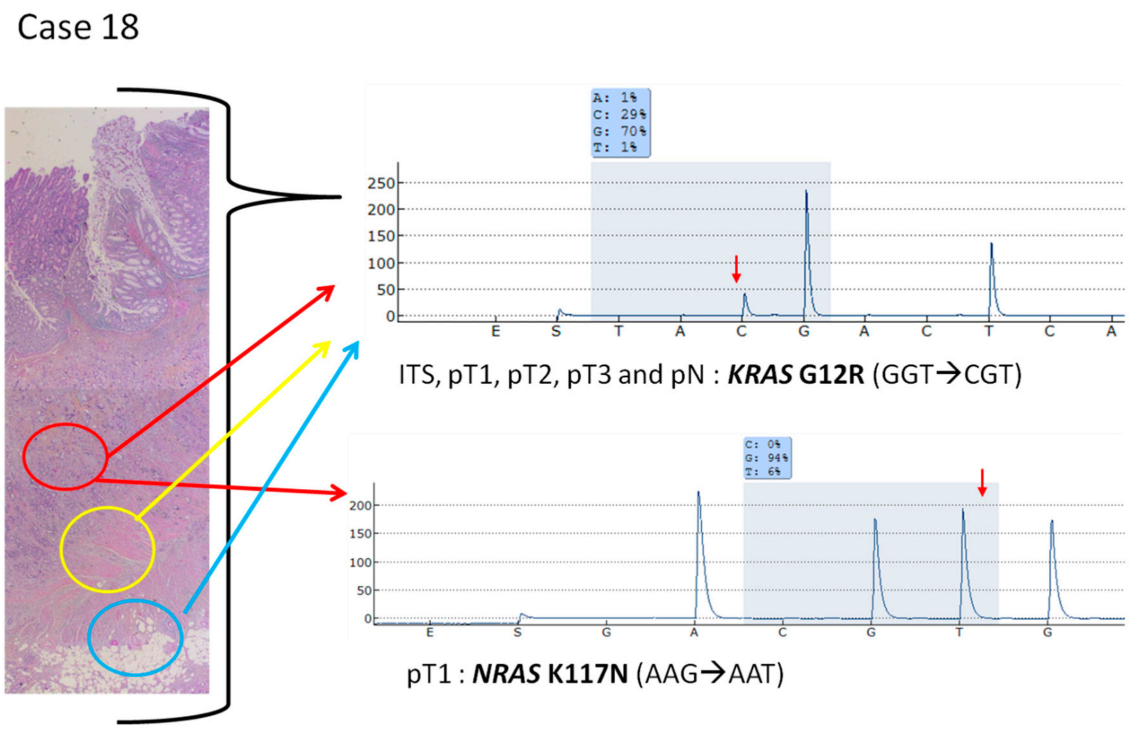


**Figure S1.** *RAS* subclones distribution for patient 18: Tumoral selection was performed according to pathological tumor node metastasis staging (pTNM) 2009 in submucosa (pT1, red arrow), in the muscular layer (pT2, yellow arrow) for pT2 tumors, selection in submucosa, in the muscular layer and in subserosa (pT3, blue arrow) for pT3 and pT4 tumors. Presence of *KRAS* MUT (p.G12R) in all selections and *NRAS* PLLM (potential low level mutation) (p.K117N) only in pT1.

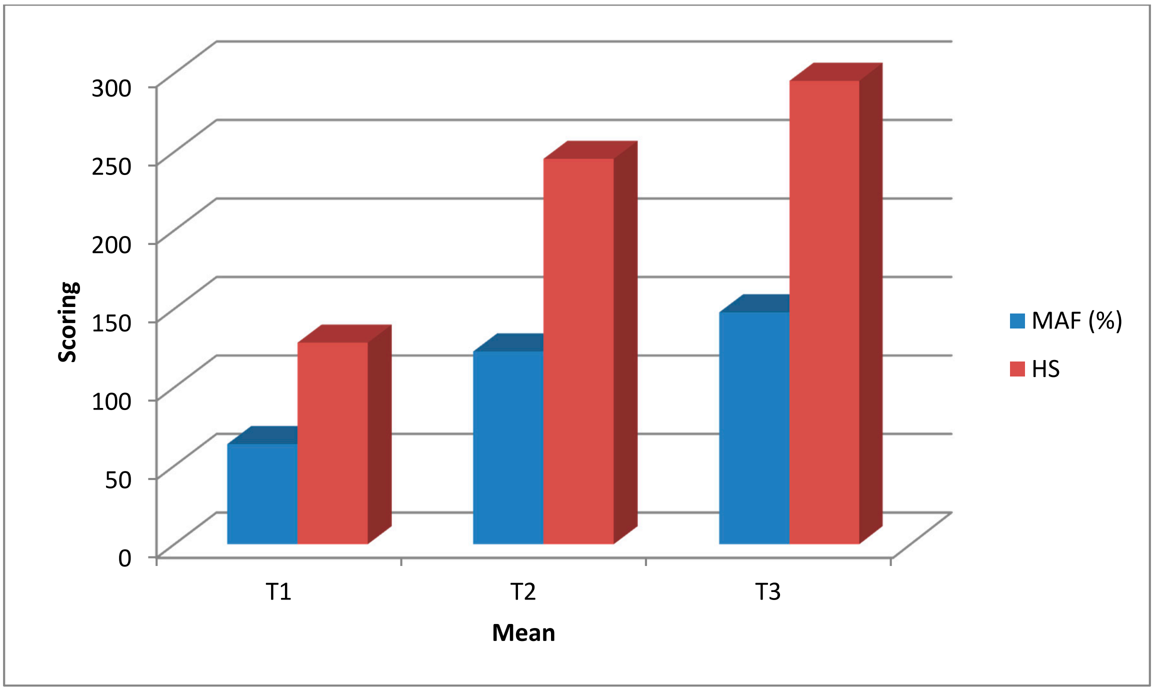


**Figure S2.** Mean heterogeneity score (HS) calculated from cases 7–9 and 13–18, which harbored the same mutation from pT1 to pT3 (Figure 1).

**Table S1.** Description of five colorectal cancers with WT mutational profile in the initial transparietal sections and spatially separated tumor areas.

| | Patients | pTNM | %TC | *KRAS* | Other Genes |
|---|---|---|---|---|---|
| No Mutation | 1 | ITS | 40 | WT | WT |
| | | pT1 | 90 | WT | WT |
| | | pT2 | 40 | WT | WT |
| | | pT3 | 30 | WT | WT |
| | | N | 70 | WT | WT |
| | 3 | ITS | 80 | WT | WT |
| | | pT1 | 60 | WT | WT |
| | | pT2 | 40 | WT | WT |
| | | pT3 | 10 | WT | WT |
| | | N | 5 | WT | WT |
| | 5 | ITS | 80 | WT | WT |
| | | pT1 | 90 | WT | WT |
| | | pT2 | 75 | WT | WT |
| | | pT3 | 25 | WT | WT |
| | 10 | ITS | 30 | WT | WT |
| | | pT1 | 20 | WT | WT |
| | | pT2 | 25 | WT | WT |
| | | pT3 | 30 | WT | WT |
| | | N | 30 | WT | WT |
| | 6 | ITS | 90 | WT | WT |
| | | pT1 | 60 | WT | WT |
| | | pT2 | 30 | WT | WT |

%TC: percentage of tumoral cells; ITS: initial transparietal section; N: metastatic lymph node; WT: wild-type; pTNM: pathological tumor node metastasis staging.

**Table S2.** Sequences of primers for *BRAF* V600E mutation detection.

| Nucleic Acid | Genomic | cDNA | Proteic | LOD | LOD + 3 | Primers | Sequences | Dispensation Order | Sequence to Analyze |
|---|---|---|---|---|---|---|---|---|---|
| *BRAF* (V600E) GAG | g.140453136 | c.1799T>A | p.V600E (Glu) | 2.3 | 5.3 | F | 5′-Biotin-TTCATGAAGACCTCACAGTA-3′ | TCGTATCTGTAG | CWCTGTAGC |
| | | | | | | R | 5′-CTTACCATCCACAAAATGGA-3′ | | |
| | | | | | | S | 5′-CCACTCCATCGAGATTT-3′ | | |
